# Fermented Total Mixed Ration Alters Rumen Fermentation Parameters and Microbiota in Dairy Cows

**DOI:** 10.3390/ani13061062

**Published:** 2023-03-15

**Authors:** Jiamei Song, Yuansheng Ma, Hengwei Zhang, Lijun Wang, Yonggen Zhang, Guangning Zhang

**Affiliations:** 1 College of Animal Science and Technology, Northeast Agricultural University, Harbin 150030, China; 2 College of Animal Science and Technology, Henan Agricultural University, Zhengzhou 450046, China

**Keywords:** fermented total mixed ration, rumen microbiota, rumen fermentation, dairy cows, 16S rRNA gene sequencing

## Abstract

**Simple Summary:**

Fermented total mixed ration (FTMR) is an effective method of preserving high-moisture byproducts. However, few studies have investigated the effect of feeding FTMR on the rumen microbiota of dairy cows. This study aimed to determine changes and interactions of ruminal microbiota and chemical parameters in dairy cows fed FTMR. Compared with unfermented total mixed ration (TMR), FTMR improved bacterial and fungal richness and diversity. Meanwhile, the ruminal acetate and NH_3_-N concentration were increased. The prediction of the metabolic function of the flora showed that FTMR could increase lipid and amino acid metabolism.

**Abstract:**

This study aimed to determine changes and interactions of ruminal microbiota and chemical parameters in dairy cows fed FTMR. Twelve multiparous Holstein dairy cows (Body weight = 616 ± 13.4 kg; day in milk = 106 ± 7.55 d; and parity = 2.31 ± 0.49; mean ± standard deviation) were divided randomly into two treatments depending on the day in milk, milk production, and parity. The two treatments were: (1) total mixed ration (TMR) and (2) FTMR. Illumina MiSeq sequencing was used to explore the changes in the ruminal microbiota. The results revealed that the bacterial and fungal diversity of the FTMR group were significantly higher than the TMR group. The predominant microbiota phyla in the bacteria and fungi showed significant differences between TMR and FTMR, as follows: *Verrucomicrobia* (*p* = 0.03) and *Tenericutes* (*p* = 0.01), *Ascomycota* (*p* = 0.04) and *Basidiomycota* (*p* = 0.04). The dominant bacterial genera in the bacteria, fungi, protozoan, and archaea that showed significant differences between TMR and FTMR were Unclassified_*Bacteroidales* (*p* = 0.02), Unclassified_RFP12 (*p* = 0.03), *Candida* (*p* = 0.0005), *Bullera* (*p* = 0.002), *Cryptococcus* (*p* = 0.007), and *Ostracodinium* (*p* = 0.01). LefSe analysis was performed to reveal the biomarker genera of the rumen microbiota community (bacteria, fungi, protozoan, and archaea) in the TMR and FTMR were the genera *Shuttleworthia*, *Ruminococcus, Cryptococcus*, *Mycosphaerella*, *Bullera*, *Candida*, and *Ostracodinium*. NH_3_-N concentration (*p* < 0.0001), total VFA concentration (*p* = 0.003), and molar proportion in total VFA of acetate (*p* = 0.01) were higher for the cows fed FTMR compared with the cows fed the TMR. Several bacterial genera showed significant correlations with rumen fermentation parameters. The genus Unclassified_*Bacteroidales* and *Bullera* were positively correlated with total volatile fatty acids (VFA) and acetate, whereas *Candida* and *Ostracodinium* showed negative correlations. Meanwhile, propionate was positively correlated with *Candida* and negatively correlated with *Bullera*. The PICRUSt functional profile prediction indicated that the xenobiotics biodegradation and metabolism, the lipid, amino acid, terpenoids, and polyketides metabolisms of the FTMR group were significantly higher than that of the TMR group. The results imply that FTMR can increase lipid and amino acid metabolism, and modulate the rumen microbiome and improve ruminal fermentation.

## 1. Introduction

Ruminants cannot effectively degrade feedstuffs independent of the vast ensemble of ruminal microbes containing bacteria, anaerobic fungi, ciliate protozoa, and archaea [1]. These microbial populations cooperate and contribute significantly to the breakdown of feed ingredients and the formation of volatile fatty acids, lactate, amino acids, lipids, and hydrogen, which are necessary for the maintenance, growth, and productivity of ruminants [2]. Regulation and optimization of the ruminal microbiota for a wide range of feeding conditions is a requirement for improving rumen function and feed efficiency [3,4,5].

Fermented total mixed ration (FTMR), which is prepared by mixing wet byproducts with dry feeds, has gradually become an effective method of preserving high-moisture byproducts [6]. The benefits of FTMR include (1) improving the population of lactic acid bacteria and concentration of lactic acid resulting in the silage pH to inhibit the growth of mold and yeast and achieving the objective of long storage time [7], (2) convenience for short-distance transportation [8], and (3) the possibility for probiotics to be delivered via silage [9]. A study has reported that additives such as lactic acid bacteria or enzymes improved the fermentation quality of FTMR [10,11,12]. *Lactobacillus buchneri* or *Lactobacillus plantarum* can improve fermentation quality and the aerobic stability of silage [10,13]. Previous studies have shown that combining inoculations with enzymes improves silage fermentation quality by degrading fiber to water-soluble carbohydrates (WSC) for lactic acid bacteria (LAB) fermentation [12,14]. In addition, feeding FTMR in livestock has been evaluated, with the results indicating that FTMR increases nutrition digestibility and volatile fatty acid content compared to non-fermented total mixed ration (TMR) [15,16]. Some studies have reported that silage-inoculated LAB can improve animal performance by increasing milk yield and feed efficiency [17,18]. Previous studies indicated that the host’s feed composition and feed intake pattern affect the rumen microbial composition [19,20,21]. Weinberg, Muck [22] demonstrated that silage inoculant LAB survived in rumen fluid in vitro. Some studies have reported silage inoculated LAB changed the ruminal bacterial community [23,24]. Different studies have shown interrelations between production variables, such as feed efficiency, milk production and composition, and the rumen microbiota [25,26]. However, there is little information about the interrelations between the rumen microbiota and rumen fermentation by feeding FTMR in dairy cows. We, therefore, hypothesized that the rumen microflora and chemical parameters would differ between FTMR and TMR. This research aimed to determine the effect of feeding FTMR on rumen fermentation parameters and microbiota in dairy cows to provide a better reference for the application of FTMR in dairy cows.

## 2. Materials and Methods

### 2.1. Animals, Experimental, and Design Diets

The animal use and care described in this study has been approved by the Animal Care and Use Committee of the Northeast Agricultural University (protocol number NEAUEC 20200236). The feeding experiment was conducted at the Songhuajiang Dairy Farm (Harbin, China). Twelve multiparous Holstein dairy cows (body weight = 616 ± 13.4 kg and day in milk (DIM) = 106 ± 7.55 d; and parity = 2.31 ± 0.49; mean ± standard deviation) were divided randomly into 2 treatments according to parity, DIM, and milk yield. The two treatments were (1) TMR (*n* = 6); (2) FTMR (*n* = 6). The experiment was conducted over 12 wk, of which the first 2 wk were used for adaptation. Cows were housed in separate stall barns that provided free access to drinking water, were fed twice daily (0630 and 1830 h) at 105% ad libitum intake, and milked twice daily at 0600 and 1800 h. The ration ingredient composition is shown in Table 1. Two diets were formulated to be isonitrogenous with a 50:50 ratio of forage to concentrate (dry matter basis) and to meet the dairy’ s requirements for nutrition [27]. All feed ingredients were mixed with a horizontal feed mixer, and lactic acid bacteria (LAB, a combination of *Lactobacillus plantarum* and *Lactobacillus buchneri* applied at a ratio of 1:1) and fibrolytic enzymes (EN, XS Biotechnology Co., Ltd., Beijing, China; 10,000 U/g) were added to the FTMR. Additives were dissolved in water and sprayed evenly over the mixture with a sprayer. The FTMR packed in polyethylene stretch film with a density of 800 kg dry matter (DM)/m^3^ were kept at ambient temperature at 16–30 ℃ for 60 days. Samples were collected after 60 days by opening the packs and testing the chemical composition of the FTMR.

### 2.2. Rumen Sample Collection and Measurements

Rumen fluid samples (approximately 200 mL) were collected by an oral stomach tube at the end of the feeding period (12 wk) approximately 3 h after the morning feeding [28]. We discarded the first 100–200 mL of fluid collected through the stomach tube to minimize the possibility that the tube would be contaminated with saliva before collecting the rumen fluid sample. Each rumen sample weighed 5 mL, which was rapidly frozen in liquid nitrogen and kept at −80 °C for later DNA extraction. The pH was measured immediately with a pH meter (PB-10; Sartorius Co., Göttingen, Germany). The samples were centrifuged under 10,000× *g*, 4 °C, 20 min, and the supernatant was separated for the further analysis of NH_3_-N and VFA. NH_3_-N concentration was measured by an alkaline phenol hypochlorite method described in 143 Solorzano. The VFA concentration was decided by gas chromatography (GC-8A; Shimadzu Corp., Kyoto, Japan) as described by Hao, Gao [29].

### 2.3. Microbial DNA Extraction, PCR Amplification, and Illumina MiSeq Sequencing

Genomic DNA was isolated from the rumen samples using the TIANamp Bacteria DNA Kit (TIANGEN, Peking, China) following the manufacturer’s protocol. PCR amplification of the extracted DNA was performed in triplicate using the Q5 High Fidelity DNA Polymerase System (New England Biolabs, Hopkinton, MA, USA). For bacterial analysis, primers 338F (5′-ACTCCTRCGGGAGGCAGCAG-3′) and 806R (5′-GGACTACCVGGGTATCTAAT-3′) were used to amplify the 16S rRNA gene’s V3-V4 region [30]. For fungal analysis, primers ITS5-F (5′-GGAAGTAAAAGTCGTAACAAGG-3′) and ITS1-R (5′-GCTGCGTTCTTCATCGATGC-3′) were used to amplify the fungal rRNA gene’s ITS1 (internal transcribed spacer region 1) [5]. For ciliate protozoal analysis, primers RP841F (5′-GACTAGGGATTGGAGTGG-3′) and Reg1302R (5′-AATTGCAAAGATCTATCCC-3′) were used to amplify the signature regions 1-2 and V3-V4 of the 18S rRNA gene [31]. The 16S rRNA gene’s V3-V4 region was amplified using primers for archaeal analysis 524F (5′-TGYCAGCCGCCGCGGTAA-3′) and 958R (5′-YCCGGCGTTGAVTCCAATT-3′). Amplification was carried out as follows: the initial denaturation step was 95 °C for 3 min; followed by 25 cycles of denaturation (95 °C for 30 s), annealing (60 °C for bacteria/54 °C for protozoa/50 °C for fungi/55 °C for archaea, for 20 s), and elongation (72 °C for 40 s); and finally, an extension step of 72 °C for 10 min. Amplicons were extracted from 2% agarose gels, purified using the DNA Gel Extraction Kit AxyPrep (Axygen Bioscience, Union City, CA, USA), using the manufacturer’s instructions, and quantified with Quant-iT PicoGreen dsDNA Assay Kit (Thermo, Carlsbad, CA, USA). Sequencing libraries were validated with an Agilent Bioanalyzer (Agilent Technologies, Palo Alto, CA, USA) and quantified with a Promega QuantiFluor (Promega, Madison, WI, USA). Finally, paired-end sequencing was performed using an Illumina MiSeq platform (Illumina, Inc., San Diego, CA, USA) at Frasergen Bioinformatics Technology, Co., Ltd. (Wuhan, China).

### 2.4. Sequencing Data Processing

Using the MiSeq platform, the sequences were processed via the opensource software pipeline QIIME (version 1.8.0) [32]. Briefly, (1) the reads with an average quality score of no less than 25 and with a length of 220–250 nt were retained; (2) only sequences with a 10 bp or greater overlap were assembled using FLASH v1.2.7 based on their overlapping sequences; (3) raw reads were quality filtered under specific filtering conditions based on QIIME’s quality control procedures (v1.8.0) to obtain high-quality clean tags. Sequences less than 160 bp in length or homopolymers longer than 8 bp were removed. Sequences were organized into operational taxonomic units (OTUs) following 97% identity using UCLUST [33], and by using USEARCH, chimeric sequences were identified and eliminated (v5.2.236, http://www.drive5.com/usearch/ accessed on 16 December 2022). The most abundant sequence in each OTU from specific libraries (bacteria, fungi, protozoa, and archaea) was selected as the “representative sequence” with an alignment against the SILVA bacterial database (version 119) [34], NCBI-nt protozoa database [35], Unite fungi ITS database (version 7.0) [36], and SILVA archaea database utilizing PYNAST [32], respectively, with the default parameters set by QIIME. Alpha diversity indices (Chao1 and Shannon) were calculated by QIIME from sparse samples using the abundance and diversity indices of the bacterial communities. Beta diversity was calculated using principal component analysis (PCA). Linear discriminant analysis (LDA) effect size (LefSe) analysis was performed on the Galaxy online analysis platform to reveal the significant ranking of abundant modules in TMR and FTMR groups [37]. A scale-effect threshold of 4.0 for the logarithmic LDA score was used to identify functional biomarkers.

### 2.5. Statistical Analyses

Statistical analysis of the data was performed by one-way analysis of variance (ANOVA) using the general linear model (GLM) of the SAS procedure (version 9.3, SAS Institute Inc., Cary, NC, USA). Pearson correlation coefficients were generated using the R software (http://www.r-project.org accessed on 16 December 2022). Significant correlations were declared at *p* ≤ 0.05.

## 3. Results

### 3.1. Rumen Fermentation Parameter

See Table 2 for the results of the rumen fermentation parameter. The total VFA content of the FTMR group was higher than that of the TMR group. Compared with cows fed TMR, cows fed FTMR had a higher molar percentage of acetate in total VFA (*p* = 0.001). The molar ratio of butyrate was lower (*p* = 0.03) in the cows fed FTMR than in those fed the TMR. Neither the molar ratio of propionate nor the proportion of acetate to propionate was significantly different between TMR and FTMR (*p* = 0.19 and *p* = 0.12, respectively).

### 3.2. Sequence, Diversity, Species Richness, and Composition of Bacterial Microbiota

**Figure 1:**
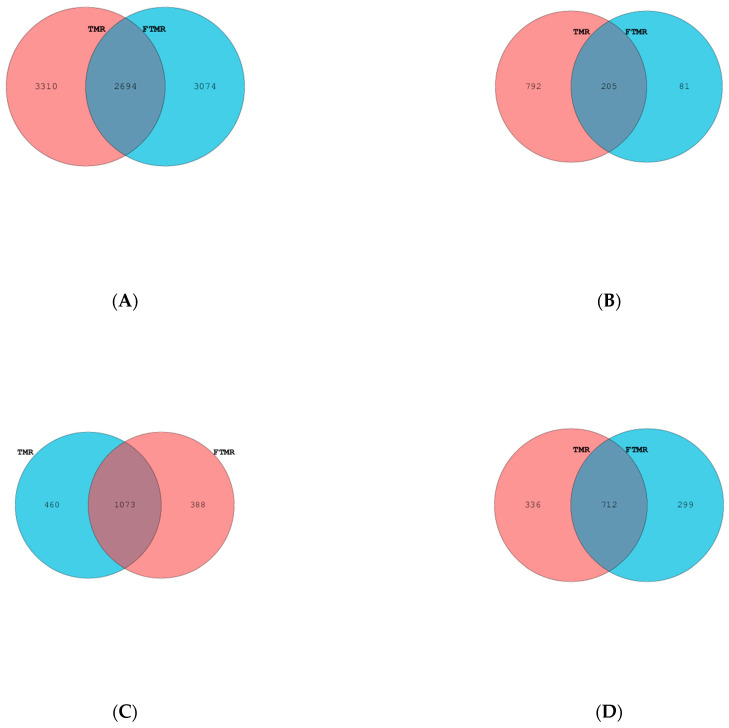
Venn diagram illustrating overlap of microbial OTUs (97% sequence identity) among treatments. Venn diagram of bacteria (**A**), ciliate protozoa (**B**), anaerobic fungi (**C**), and archaea (**D**) OTUs. TMR, total mixed ration; FTMR, fermentation total mixed ration.

**Figure 2:**
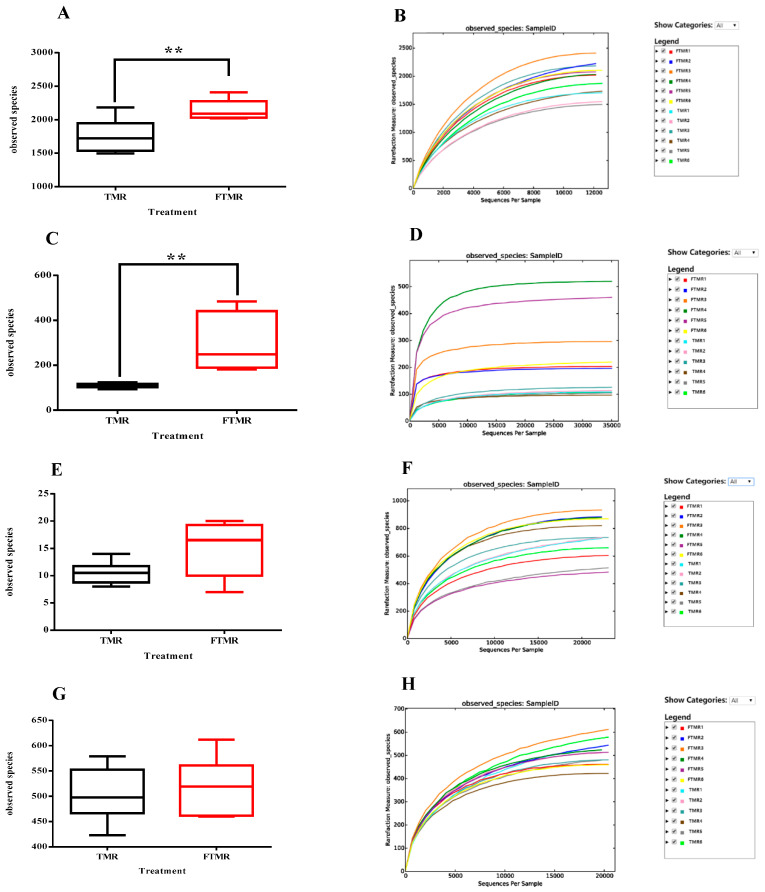
Boxplot and rarefaction curves of operational taxonomic units (OTUs). Bacterial (**A**), anaerobic fungi (**C**), protozoan (**E**), and archaea (**G**) boxplot represent the number of observed OTUs. The *x*-axis shows the observed species (OTUs), and the *y*-axis shows different groups (between TMR and FTMR). The OTU similarity threshold of 97% was considered. Boxes represent the interquartile range (IQR) between the first and third quartiles (25th and 75th percentile, respectively), and whiskers indicate the extremes of the data. ** *p* < 0.01 (Student’s t-test). Bacterial (**B**), anaerobic fungi (**D**), protozoan (**F**), and archaea (**H**) rarefaction curves of OTUs. The *x*-axis shows the number of valid sequences per sample, and the *y*-axis shows the observed species (OTUs). Each curve in the graph indicates a different sample and is shown in a different color. The longer the curve, the higher the sequencing depth and the more OTUs are observed. The flatter the curve, the more the sequencing results reflect the diversity contained in the current sample. TMR, total mixed ration; FTMR, fermented total mixed ration.

A total of 367,333 high-quality bacterial sequences were obtained from 6 TMR and 6 FTMR rumen samples, with an average of 27284–33184 (Mean = 30611 ± 484) sequences per sample. In total, 11,772 OTUs were identified, of which 2694 OTUs were found in two treatment groups (Figure 1A). The Good’s coverage in this study was close to 99%. Within sample groups, core bacterial communities are defined by the taxa shared by all individuals within those groups. As shown in Figure 2A, the observed species (OTUs) in TMR and FTMR. Cows fed FTMR demonstrated a higher species (*p* < 0.01) than cows fed TMR. As sequencing depth increases, the rarefaction curve at 97% similarity reveals an increase in OTU numbers. The ends of the sparsity curve tended to be flat with the increase in sequencing data (Figure 2B).

**Figure 3:**
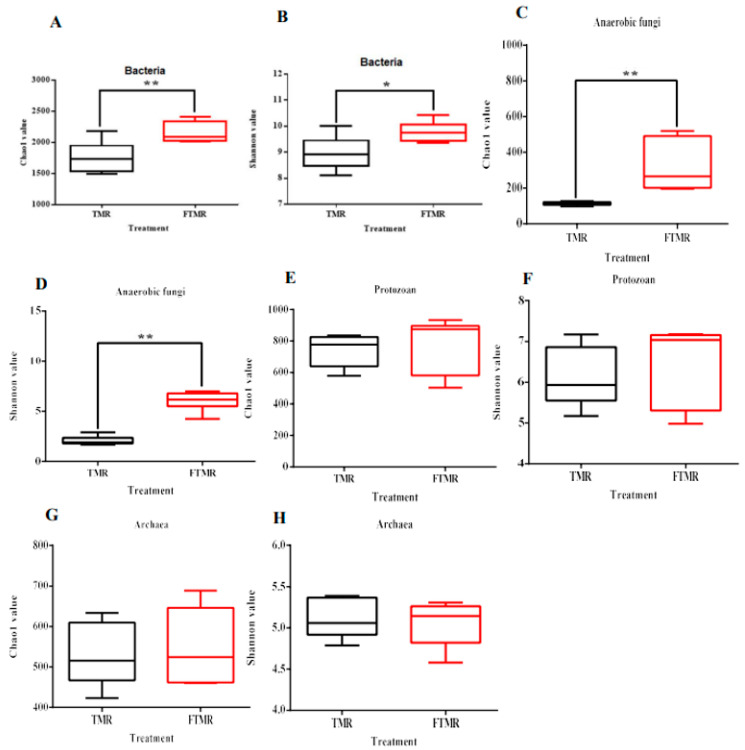
Changes in ruminal microbial richness and diversity between TMR and FTMR. Bacterial (**A**), anaerobic fungi (**C**), protozoan (**E**), and archaea (**G**) richness are estimated by the Chao1 value. Bacterial (**B**), anaerobic fungi (**D**), protozoan (**F**), and archaea (**H**) diversity are estimated by the Shannon index. Boxes indicate the interquartile range (IQR) between the first and third quartiles (25th and 75th percentile, respectively), and whiskers indicate the extremes of the data. Boxes with the different asterisk above their whiskers are significantly different (* *p* < 0.05 and ** *p* < 0.01, respectively) among treatments. TMR, total mixed ration; FTMR, fermentation total mixed ration.

**Figure 4:**
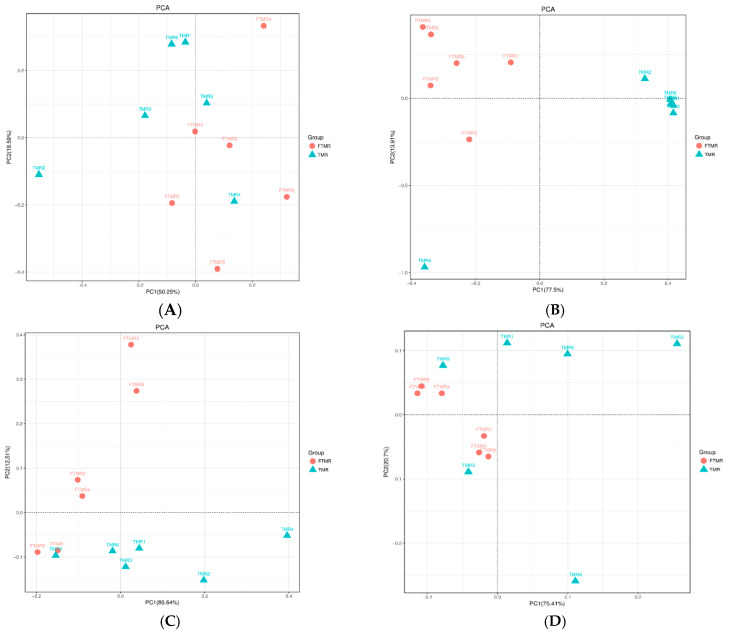
PCA of rumen microbial communities. Each point represents a sample, and different colors represent different groups. The closer the distance between two points, the less the difference in microbial community structure between the two samples. Weighted PCA by ruminal bacteria (**A**), anaerobic fungi (**B**), protozoan (**C**), and archaea (**D**). TMR, total mixed ration; FTMR, fermented total mixed ration.

Analysis of the alpha diversity (Chao1 and Shannon) (Figure 3A,B) showed that FTMR-fed cows had higher Chao 1 and Shannon values (*p* < 0.05 and *p* < 0.01, respectively) than TMR-fed cows (Figure 3A,B). PCA analyses showed that rumen bacterial communities differed between TMR and FTMR (Figure 4A). TMR and FTMR can be largely separated at the OTU level.

**Figure 5:**
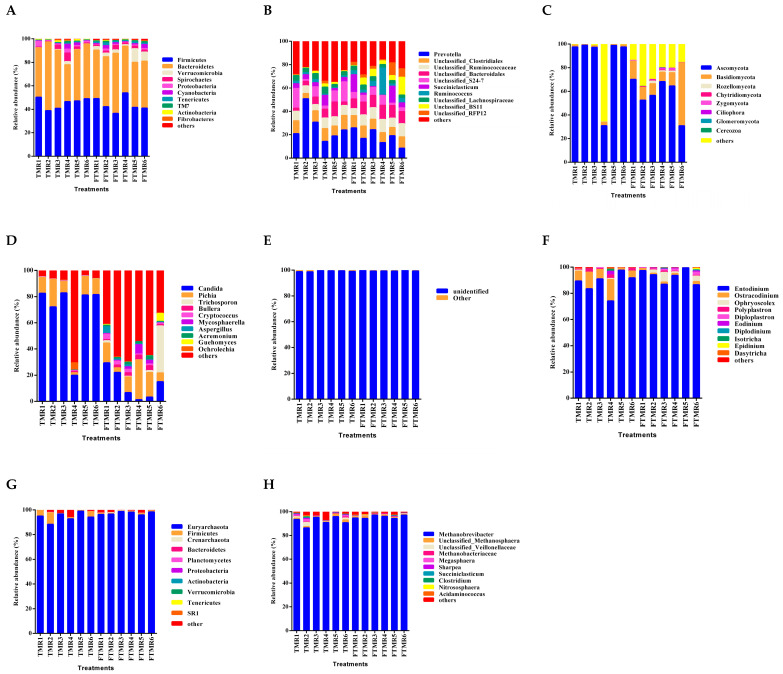
Relative abundance of microbial phyla and genera in the TMR and FTMR. The abundance of the bacteria phyla (**A**), anaerobic fungi (**C**), protozoa (**E**), and archaea (**G**); abundance of genera of bacteria (**B**), anaerobic fungi (**D**), protozoa (**F**), and archaea (**H**). Only the top 10 phyla and genera in abundance are shown in the figure; other species were combined as “others”. TMR, total mixed ration; FTMR, fermentation total mixed ration.

In total, 17 different bacterial phyla were recognized, and the top 10 phyla were identified based on the relative abundance of rumen bacteria present in Figure 5A. The relative abundance of bacterial phyla higher than 1% was considered predominant. Among these phyla, the predominant bacteria in the TMR and FTMR firstly with *Firmicutes*, followed by *Bacteroidetes*, *Verrucomicrobia*, *Spirochaetes*, *Proteobacteria*, *Cyanobacteria*, and *Tenericutes*. An analysis of the phyla showing significant differences between TMR and FTMR were *Verrucomicrobia* (*p* = 0.03) and *Tenericutes* (*p* = 0.01) (Table 3). By analyzing the microbiota composition, 143 bacterial taxa were identified at the genus level, and 53 genera were observed in all samples. The relative abundance of the top 10 genera of rumen bacteria is displayed in Figure 5B. The bacterial genera with relative abundance >1% were considered to be dominant. Among these genera, the dominant bacteria in the TMR and FTMR were *Prevotella*, Unclassified_*Clostridiales*, Unclassified_*Ruminococcaceae*, Unclassified_*Bacteroidales, Succiniclasticum, Ruminococcus*, and Unclassified_*Lachnospiraceae*. The results showed significant genera differences between TMR and FTMR were Unclassified_*Bacteroidales* (*p* = 0.02) (Table 3).

**Figure 6:**
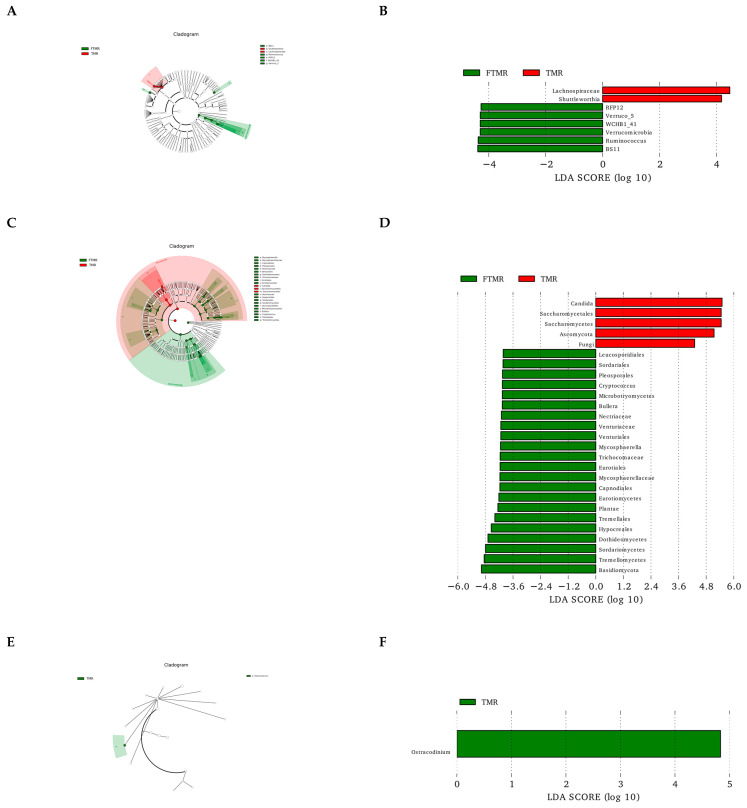
The LDA effect size (LefSe) analysis. The cladogram diagram shows the microbial genera with significant differences in the TMR and FTMR groups (from phylum to genus level). The cladogram diagram of bacteria (**A**), anaerobic fungi (**C**), protozoa (**E**); species with significant differences that have a linear discriminant analysis (LDA) score greater than the estimated value (**B**,**D**,**F**). The histogram of bacteria (**B**), anaerobic fungi (**D**), and protozoa (**F**). Significant differences are defined as *p* < 0.05 and LDA score > 4.0. The histogram length represents the LDA score, i.e., the degree of influence of the species with the significant differences between the different groups. TMR, total mixed ration; FTMR, fermentation total mixed ration.

An analysis of LefSe was conducted to uncover the rumen bacteria community’s biomarker genera in the TMR and FTMR in Figure 6A,B. The TMR group’s most differentially abundant bacterial taxa belonged to the genera *Shuttleworthia*, whereas the genera *Ruminococcus* was more abundant in the FTMR group. The genera *Shuttleworthia* and *Ruminococcus* were the most differentiated among communities, with an absolute LDA score factor of four.

### 3.3. Sequence, Diversity, Species Richness, and Composition of Anaerobic Fungal Microbiota

A total of 586,464 high-quality rumen fungi sequences were acquired from six TMR, and six FTMR rumen samples, with 40,207–58,811 (Mean = 48,872 ± 1670) analyzed sequences (Mean length = 238.9 bp) in each sample. There were 1283 OTUs identified, among which 205 OTUs were found in two treatment groups (Figure 1B). The Good’s coverage in this study was close to 99%. Figure 2C shows the observed species (OTU) in TMR and FTMR, and the number of species in the TMR was lower than in the FTMR. FTMR-fed cows demonstrated a higher observed species (*p* < 0.01) than cows fed TMR. As sequencing depth increases, the rarefaction curve at 97% similarity reveals an increase in OTU numbers. The ends of the sparsity curve tended to be flat with the increase in sequencing data (Figure 2D).

Analysis of the alpha diversity (Chao1 and Shannon) (Figure 3C,D) and Beta diversity (PCA) (Figure 4B) of the rumen fungi revealed variance in the microbial community. FTMR-fed cows demonstrated higher Chao 1 and Shannon values than those TMR-fed cows. The PCA results demonstrated the diversity of the rumen fungi community and showed that TMR and FTMR could substantially be distinguished from one another at the OTU level.

In total, seven rumen fungal phyla were confirmed across all the samples. The relative abundance of all shared phyla in rumen fungi is displayed in Figure 5C. The relative abundance of fungal phyla higher than 1% was considered predominant. Among these phyla, the dominant rumen fungi in the TMR and FTMR were *Ascomycota* and *Basidiomycota*. An analysis of the phyla showing significant differences between TMR and FTMR were *Ascomycota* (*p* = 0.04) and *Basidiomycota* (*p* = 0.04) (Table 3). Through analysis of microbiota composition, this study identified 244 fungal taxa and 100 genera observed across all samples. The relative abundance of the shared top 10 genera in rumen fungi is displayed in Figure 5D. The relative abundance of fungal genera with >1% was considered predominant. Among these genera, the dominant fungi in the TMR and FTMR were *Candida*, *Pichia*, *Mycosphaerella*, *Trichosporon*, *Bullera*, *Ruminococcus*, and *Cryptococcus*. A significant difference was found between TMR and FTMR as follows, *Candida* (*p* = 0.0005), *Bullera* (*p* = 0.002), and *Cryptococcus* (*p* = 0.007) (Table 3).

LefSe analysis was conducted to uncover the biomarker genera of the rumen anaerobic fungi community in the TMR and FTMR in Figure 6C,D. The largest difference of fungi in the TMR group belonged to the genera *Candida*. *Cryptococcus*, *Mycosphaerella*, and *Bullera* were more abundant than that in the FTMR group. The genera *Cryptococcus*, *Mycosphaerella*, *Bullera*, and *Candida*, were the most differentiated among communities, with an absolute LDA score factor of 4.

### 3.4. Sequence, Diversity, Species Richness, and Composition of Ciliate Protozoal Microbiota

In total, 370,861 high-quality rumen protozoan sequences were obtained from 6 TMR and 6 FTMR rumen samples, and 27,043–35,796 (Mean = 30,905 ± 858) sequences (Mean length = 350.1 bp) were analyzed in each sample. A total of 2994 OTUs were identified, of which 1073 OTUs were found in two treatment groups (Figure 1C). Good’s coverage in this study was close to 99%. The core rumen protozoa communities are the bacterial population common to all group members. Figure 2E showed the species (OTUs) observed in the TMR and FTMR (OTU). The cows fed FTMR had no significant differences in observed species (*p* > 0.05) to cows fed TMR. As sequencing depth increases, the rarefaction curve at 97% similarity reveals an increase in OTU numbers. The ends of the sparsity curve tended to be flat with the increase in sequencing data (Figure 2F).

Analysis of the alpha diversity (Chao1 and Shannon) (Figure 3E,F) and Beta diversity (PCA) (Figure 4C) of the rumen protozoan revealed variance in the microbial community. There were no significant differences in Chao 1 and Shannon values between cows fed FTMR and those fed TMR (*p* > 0.05 and *p* > 0.05, respectively). PCA analyses showed that the rumen protozoan community differed between TMR and FTMR. TMR and FTMR can be largely separated at the OTU level.

Rumen protozoal phyla were not identified across all the samples (Figure 5E). By analyzing the microbiota composition, 13 protozoan taxa were identified, and 10 genera were observed across all samples. The relative abundance of shared the top 10 genera in rumen protozoan is displayed in Figure 5F. The relative abundance of protozoal genera higher than 1% was considered predominant. Among these genera, the dominant protozoan in the TMR and FTMR were *Entodinium*, *Ostracodinium*, and *Ophryoscolex*. A significant difference was found between TMR and FTMR in the abundances of *Ostracodinium* (*p* = 0.01) (Table 3).

LefSe analysis was conducted to uncover the biomarker genera of the rumen ciliate protozoan community in the TMR and FTMR in Figure 6E,F. The TMR group’s most differentially abundant ciliate protozoan taxa belonged to the genera *Ostracodinium*. No rumen ciliate protozoan community was not observed in the FTMR group. The genera *Ostracodinium* was the most differentiated among communities, with an absolute LDA score factor of 4.

### 3.5. Sequence, Diversity, Species Richness, and Composition of Archaeal Microbiota

A total of 389140 high-quality sequences in rumen archaea were acquired from six TMR and six FTMR rumen samples, and 27,065–37,010 sequences (Mean = 32,428 ± 999) were analyzed (Mean length = 287.3 bp) in each sample. There were 2059 OTUs identified, among which 712 OTUs were found in two treatment groups (Figure 1D). The Good’s coverage in this study was close to 99%. The taxa that all individuals shared were considered core rumen archaea communities within each sample group. The observed species (OTUs) in TMR and FTMR are shown in Figure 2G. The cows fed FTMR had no significant differences in observed species (*p* > 0.05) to cows fed TMR. As sequencing depth increases, the rarefaction curve at 97% similarity reveals an increase in OTU numbers. The ends of the sparsity curve tended to be flat with the increase in sequencing data (Figure 2H).

Analysis of the alpha diversity (Chao1 and Shannon) (Figure 3G,H) and Beta diversity (PCA) (Figure 4D) of the rumen archaea revealed variance of the microbial community. There were no significant differences in Chao 1 and Shannon values between cows fed FTMR and those fed TMR (*p* > 0.05 and *p* > 0.05, respectively). PCA analyses showed that the rumen archaea community differed between TMR and FTMR. TMR and FTMR can be largely separated at the OTU level.

In total, seven rumen archaeal phyla were confirmed across all samples. The relative abundance of shared phyla in rumen archaea is displayed in Figure 5G. The relative abundance of archaeal phyla higher than 1% was considered predominant. Among these phyla, the dominant rumen archaea in the TMR and FTMR were *Euryarchaeota* and *Firmicutes*. There were 45 archaeal taxa identified at the genus level, and 16 genera were observed in all samples. The relative abundance of shared the top 10 genera in rumen archaea is displayed in Figure 5H. The phyla with greater than 1% relative abundance were classified as dominant phyla. Among these genera, the dominant archaea in the TMR and FTMR were *Methanobrevibacter* and Unclassified*_Methanosphaera*. An analysis of the phyla showing significant differences between TMR and FTMR was *Methanobrevibacter* (*p* = 0.046) (Table 3). LefSe analysis revealed the biomarker genera for the rumen archaeal community in the TMR, and FTMR was not shown in the rumen.

### 3.6. The Correlations between the Relative Abundance of Predominant Ruminal Microbiota and Rumen Fermentation Parameter

A correlation analysis was performed to examine the relationship between the predominant ruminal microbiota and rumen fermentation parameters (Table 4). In rumen bacteria, the relative abundance of Unclassified_*Bacteroidales* had a positive association (*p* < 0.05) with total VFA and acetate molar proportion. In rumen fungi, total VFA, acetate, and propionate molar proportion were positively correlated with (*p* < 0.05) the relative abundance of *Bullera*, whereas evenness showed a negative correlation with (*p* < 0.05) the relative abundance of *Candida*. The relative abundance of *Cryptococcus* was positively related to (*p* < 0.05) the NH_3_-N and acetate molar proportion. In rumen protozoa, the relative abundance of *Ostracodinium* was negatively correlated with (*p* < 0.05) NH_3_-N, total VFA, and acetate molar proportion. In rumen archaea, the relative abundance of *Methanobrevibacter* and butyrate molar proportion displayed a negative correlation.

### 3.7. Prediction of Different Treatment Functions of Rumen Bacteria

**Figure 7:**
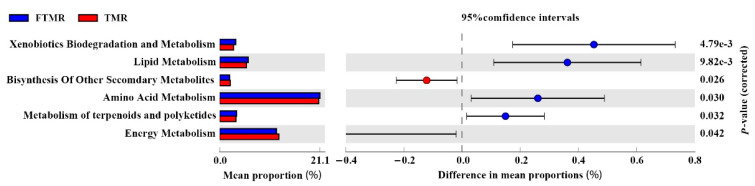
Phylogenetic Investigation of Communities by Reconstruction of Unobserved States (PICRUSt) analysis. Variance analysis of the Kyoto Encyclopedia of Genes and Genomes (KEGG) metabolic pathways in the second level. The graphs show the abundance ratio of different functions in two groups of samples. The middle shows the difference between the proportions of functional abundance in the 95% confidence interval, and the value at the rightmost is the *p*-value. *p* < 0.05 represents the significant difference. TMR, total mixed ration; FTMR, fermentation total mixed ration.

The functional gene composition between FTMR and TMR was inferred using the PICRUSt software by comparing the species composition information from 16S sequencing data. Differential analysis of KEGG metabolic pathways was used to analyze the differences and changes in the functional genes of microbial communities in the metabolic pathways between different groups, thereby evaluating metabolic changes in different rumen samples in response to environmental changes. In the present study, at KEGG level 2, a total of six KEGG metabolic pathways enriched significant differences between FTMR and TMR (*p* < 0.05) (Figure 7). In comparison with the TMR group, the xenobiotics biodegradation and metabolism, lipid metabolism, amino acid metabolism, and metabolism of terpenoids and polyketides of the FTMR group were higher (*p* < 0.05) (Figure 7). However, compared to the FTMR group, the biosynthesis of other secondary metabolites and energy metabolism of the TMR group were higher (*p* < 0.05), indicating that FTMR increases lipid and amino acid metabolism.

## 4. Discussion

### 4.1. Rumen Fermentation Parameter

The current study looked at the interactions between rumen fermentation parameters and ruminal microbiota when TMR or FTMR were fed to animals. The result of this study was were in accordance with Cao, Takahashi [15], who also saw higher concentrations of NH_3_-N when FTMR was fed to sheep compared to non-fermented TMR. Ruminal NH_3_-N is the major source of N for microbial growth [38,39,40]. Cao, Takahashi [15] observed higher digestible energy in sheep that were fed fermented TMR compared with those fed nonfermented TMR. Thus, cows fed FTMR diets may have increased microbial crude protein synthesis and thus affected milk protein content [41,42]. However, this hypothesis needs to be tested by further studies. The total VFA concentration of the FTMR diet was higher than that of the TMR diet. In this study, the total VFA concentration agreed with Cao, Takahashi [15] and Miyaji, Inoue [43]. This suggests that the consumption of small-molecule carbohydrates can increase the VFA concentration. Although the increased VFA concentration in cows fed the FTMR diet, the difference in pH between the two diets was not significant. FTMR promoted the absorption of VFA by rumen epithelium [15]. The acetic acid concentration in cows fed the FTMR diet was due to improved in situ degradation of neutral detergent fiber (NDF) and silage processing in this study. Dairy cows fed easily fermentable carbohydrates also show a high propionic acid concentration, which agrees with the previous results [15]. In addition, ruminal lactate-utilizing bacteria play a key role in maintaining normal rumen fermentation, reducing the accumulation of lactate, and maintaining a normal rumen environment by converting lactate into propionate [15,44]. The ratio of acetate to propionate in both diets was far greater than 2.5, considered the threshold for milk fat reduction [45].

### 4.2. Diversity, Species Richness, and Composition of Bacterial, Anaerobic Fungal, Ciliate Protozoal, and Archaeal Microbiota

In this study, our results showed that cows fed FTMR positively affected bacterial richness (e.g., Chao1 value) and diversity (e.g., Shannon index). This was possibly due to this study confirming that providing more readily fermentable carbohydrates decreases bacterial richness and diversity. In the present study, regardless of which diet, *Firmicutes* and *Bacteroidetes* were the most dominant bacterial phylum in the cow rumen, confirmed by many former researchers [46,47,48]. The relative abundance of *Verrucomicrobia* significantly increased in the cows fed FTMR. This was observed in the rumen microbiota of goats after 4 weeks of medium non-fiber carbohydrates feeding [49]. Previous research indicated that *Verrucomicrobia* plays an important role in acquiring immune tolerance in mice gut [50,51]. However, further studies are needed to investigate the role of *Verrucomicrobia* in the dairy gut.

In this study, *Prevotella*, Unclassified_*Clostridiales*, Unclassified_*Ruminococcaceae*, Unclassified_*Bacteroidales*, *Succiniclasticum*, *Ruminococcus, and* Unclassified_*Lachnospiraceae* were the major bacteria, which were recognized by most past studies [48,52,53,54]. The relative abundance of Unclassified_*Bacteroidales* significantly increased the cows fed FTMR over those fed TMR. LefSe analysis showed that biomarkers between the TMR and FTMR groups on the genus level constitute *Shuttleworthia* and *Ruminococcus* showed significant differences.

In the current study, feeding FTMR to cows positively affected the fungal abundance and diversity, probably because *Ascomycota* and *Basidiomycota* were the most dominant fungal phylum in cow rumen, whereas previous studies have reported similar findings [5,30]. Possibly because of the primers used for sequencing [30], several ruminal fungi were uncommon in former research [55,56]. Most of these phyla had been detected from marine samples (Jones et al., 2015) and were seldom found in ruminants. In the rumen ecosystem, it has yet to be discovered what metabolic and functional significance. Therefore, the reasons for decreasing *Ascomycota* and increasing *Basidiomycota* in FTMR-fed animals still need to be clarified. The differences in *Candida*, *Bullera*, and *Cryptococcus* genera between FTMR and TMR groups were judged to be significant. LefSe analysis showed that biomarkers indicating significant differences between the TMR and FTMR groups at the genus level included *Cryptococcus*, *Mycosphaerella*, *Bullera*, and *Candida*.

*Entodinium*, *Ostracodinium*, and *Ophryoscolex* were the most abundant bacterial community in the present study, which was similar to the previous studies [30,55,57,58]. Zhang, Shi [30] and Henderson, Cox [47] observed that *Ostracodinium* as the predominant bacteria in the rumen decreased linearly as the percentage of dietary concentrate level increased. Sandra, Henning [56] found that *Ostracodinium* was the major genera in ruminants fed silage. However, a relatively low abundance of *Ostracodinium* was observed in the FTMR group. LefSe analysis showed that biomarkers indicating significant differences between the TMR and FTMR groups at the genus level included *Ostracodinium. Ostracodinium* has a large capacity to degrade cellulose [58], explaining its relatively low abundance in FTMR, where there was a lower fiber content because of the addition of lactobacillus and fibrolytic enzyme [12]. Moreover, a previous study found that the total VFA and NH_3_-N concentrations were increased when sheep were fed an FTMR involved in whole grain rice and rice bran [15]. The relatively low abundance of *Ostracodinium* can be associated with the increased fermentation rate of FTMR and the concomitant decrease in pH [59,60].

In this study, *Euryarchaeota*, *Methanobrevibacter*, and Unclassified_*Methanosphaera* were the most predominant archaeal phylum and genus in the rumen, which was found in most previous studies [30,47]. In accordance with a comparative study [61,62], *Methanobrevibacter* was hydrogenotrophic and produced CH_4_ by utilizing hydrogen or formate in ruminants. Total VFA (Table 2) in the rumen possibly resulted in the relatively high abundance of *Methanobrevibacter* in the FTMR group. At the same time, the relatively high abundance of *Methanobrevibacter* in the FTMR group revealed that the cows fed FTMR possibly increased CH_4_ content, which was different from the observation of Cao, Takahashi [15]. Cao, Takahashi [15] observed that FTMR containing whole-crop rice and rice bran decreased ruminal methane emission, possibly because lactate-utilizing bacteria convert lactic acid or pyruvic acid to propionic acid in sheep. However, no increase in propionic acid and lactate-utilizing bacteria was observed in this study.

### 4.3. Correlations between the Relative Abundance of Predominant Ruminal Microbiota and Rumen Fermentation Parameter

The study found that Unclassified*-Bacteroidales* were positively correlated with acetate, an important end product of rumen metabolism. Zhu et al. [63] found that an increase in the abundance of acetate producing microbiota led to an increase in acetate content. Therefore, this result indicated that Unclassified*-Bacteroidales* enabled acetate production. *Candida* and *Bullera* belong to the yeast; however, we observed an opposite relationship with total VFA, acetate, and propionate. These results agree with the relative abundance of *Candida* and *Bullera* as well as total VFA, acetate, and propionate. *Ostracodinium* was negatively correlated with NH_3_-N, total VFA, and acetate. Moreover, the relative abundance of *Ostracodinium* in the FTMR group was significantly lower than in the TMR group. A previous study showed that *Ostracodinium* was not detected when fed a high-concentrate diet [60]. These results indicated that FTMR contained more soluble carbohydrates and thus promoted more VFA production from rumen fermentation. *Methanobrevibacter* was negatively correlated with butyrate. *Methanobrevibacter* is a major intestinal methanogenic archaeon that can grow on H_2_/CO_2_ or formate. Meanwhile, some microbes can convert H_2_ and CO_2_ to butyrate in the gut [64]. Overall, the interactive analysis provided novel insights to elucidate the symbiotic relationships between rumen microbiota and metabolism.

### 4.4. Function Prediction of Rumen Bacteria

PICRUSt was used to understand the metabolic functions of rumen bacteria further. PICRUSt analysis indicated that comparing two dietary treatments simultaneously; we investigated the functional genes required for biodegradation and metabolism of xenobiotics, lipid metabolism, amino acid metabolism, and metabolism of terpenoids and polyketides of the FTMR group was significantly higher than that of the TMR group. This suggests that FTMR can increase lipid and amino acid metabolism. In addition, studies show that the lipid metabolism and amino acid metabolism of the high-feed efficiency group were higher than those of the low-feed efficiency group [65]. Meanwhile, a meta-analysis of 23 in vivo studies also demonstrated an improvement in feed efficiency, fat, and protein-corrected milk following supplementation with Agolin oil during periods longer than one month [66]. In this study, the lipid metabolism and amino acid metabolism of the FTMR group were significantly higher than that of the TMR group, which indicated that FTMR could increase feed efficiency. The improvement in feed efficiency will reduce feed resource consumption and lower feeding costs [67]. Therefore, feeding FTMR may be a better alternative strategy to improve the performance of lactating cows. Although the relatively high abundance of *Methanobrevibacter* in the FTMR group may have increased CH4 content, the higher feed efficiency may have mitigated methane emissions.

## 5. Conclusions

In summary, through high-throughput sequencing, the present study investigates impacts of FTMR on ruminal microbial communities. Compared with unfermented TMR, FTMR improved bacterial and fungal richness and diversity. The relative abundances of Unclassified*_Bacteroidales*, *Bullera*, and *Cryptococcu*s were significantly increased, whereas *Ostracodinium* decreased in FTMR compared to TMR. Meanwhile, the ruminal acetate and NH_3_-N concentration were increased. Our results also showed positive correlations of total VFA, acetate with Unclassified *Bacteroidales* and *Bullera*, but negative correlations with *Candida* and *Ostracodinium*; propionate was positively correlated with *Candida* and negatively with *Bullera*. The PICRUSt functional profile prediction showed that the FTMR could increase lipid and amino acid metabolism. However, further experiments are required to gain a deeper understanding of the mechanisms involved.

## Figures and Tables

**Table 1:** Ingredients and chemical composition of two treatment diets.

Item	Treatment	
	TMR ^1^	FTMR ^1^
Ingredient, % of DM		
Alfalfa hay	11.8	11.8
Wet corn gluten feed	6.9	6.9
Corn stover	3.4	3.4
Corn silage	27.8	27.8
Ground corn	24.6	24.6
Soybean meal	9.8	9.8
Cottonseed meal	4.9	4.9
DDGS	7.4	7.4
Expanded soybean	1.0	1.0
Premix ^2^	2.5	2.5
Chemical composition		
DM, % of DM	48.0	46.2
CP, % of DM	17.5	19.1
NDF, % of DM	38.3	35.7
ADF, % of DM	21.5	20.5
NFC ^3^, % of DM	35.3	34.8
Starch, % of DM	21.7	22.4
NE_L_^4^, Mcal/kg of DM	1.61	1.64
Fermentation profile		
pH	6.01	4.69
Lactic acid, % of DM	0.63	8.48
Acetic acid, % of DM	0.64	2.23
NH_3_-N, % of Total N)	2.65	5.28

^1^ TMR = total mixed ration; FTMR = fermented total mixed ration. ^2^ Premix contained (DM basis) 99.07% of ash, 14.27% of Ca, 5.42% of P, 4.96% of Mg, 0.05% of K, 10.67% of Na, 2.98% of Cl, 0.37% of S, 11 mg/kg of Co, 577 mg/kg of Cu, 4858 mg/kg of Fe, 51 mg/kg of I, 1806 mg/kg of Mn, 13 mg/kg of Se, 1694 mg/kg of Zn, 115,240 IU/kg of vitamin A, 46,100 IU/kg of vitamin D, and 576 IU/kg of vitamin E. ^3^ NFC = 100 − % NDF − % CP − % ether extract − % ash. ^4^ Calculated based on Ministry of Agriculture of P.R. China (MOA, 2004).

**Table 2:** Rumen fermentation parameters between the TMR and FTMR in dairy cows.

Item	Treatment ^1^		SEM ^2^	*p*-Value
	TMR	FTMR		
pH	6.50	6.41	0.10	0.55
NH_3_-N, mg/dL	13.6	21.2	0.80	<0.0001
Total VFA, mM	78.0	90.6	2.21	0.003
Molar proportion, mmol/100 mmol				
Acetate	50.0	62.3	1.97	0.001
Propionate	18.0	19.5	0.79	0.19
Butyrate	10.04	8.74	0.35	0.03
Acetate:propionate	2.80	3.22	0.17	0.12

^1^ TMR, total mixed ration; FTMR, fermented total mixed ration. ^2^ SEM, standard error of the mean.

**Table 3:** The diversity of main rumen microbiota that significantly changed in the phyla and genera level between the TMR and FTMR in dairy cows. (accounting for ≥1% of the total sequences in the abundance of top 10 genera in at least one of the samples).

Item	Phyla and Genera Level	Treatment ^1^		SEM ^2^	*p*-Value
		TMR	FTMR		
Bacteria	Unclassified_*Bacteroidales*	6.00	10.47	1.16	0.02
	*Verrucomicrobia*	0.60	4.87	1.17	0.03
	*Tenericutes*	0.62	1.35	0.18	0.01
Anaerobic fungi	*Ascomycota*	86.8	57.0	8.97	0.04
	*Candida*	69.9	12.9	7.87	0.0005
	*Basidiomycota*	1.52	18.1	5.03	0.04
	*Bullera*	0.22	2.33	0.36	0.002
	*Cryptococcus*	0.25	2.17	0.40	0.007
Protozoan	*Ostracodinium*	8.37	1.45	1.55	0.01
Archaea	*Methanobrevibacter*	92.0	95.4	1.08	0.05

^1^ TMR, total mixed ration; FTMR, fermentation total mixed ration. ^2^ SEM, standard error of the mean.

**Table 4:** Correlations between the predominant ruminal microbiota ^1^ affected by treatments and rumen fermentation parameters.

Genus	PH	NH_3_-N (mg/dL)	Total VFA (mmol/L)	Acetate (%)	Propionate (%)	Butyrate (%)
Unclassified_*Bacteroidales*	−0.02	0.32	0.65 *	0.63 *	0.43	−0.41
*Candida*	0.17	−0.42	−0.71 *	−0.66 *	−0.66 *	0.27
*Bullera*	−0.06	0.55	0.71 *	0.66 *	0.59 *	−0.17
*Cryptococcus*	−0.3	0.88 *	0.56	0.64 *	0.25	−0.42
*Ostracodinium*	0.19	−0.62 *	−0.76 *	−0.77 *	−0.42	0.46
*Methanobrevibacter*	−0.27	0.51	0.22	0.36	−0.10	−0.75 *

^1^ Only the significantly affected ruminal microbiota (at the genera level) with a mean relative abundance >1% in the top 10 genera were considered in the correlation. * Significant (*p* ≤ 0.05) Pearson correlation coefficient.

## Data Availability

The Illumina sequencing raw data for our samples have been submitted to the NCBI Sequence Read Archive (SRA) under accession number: PRJNA870710. All raw sequence data in sequencing data processing were deposited to NCBI Sequence Read Archive under accession number SRP089832.

## References

[1] Hobson P.N., Stewart C.S. (1997). The Rumen Microbial Ecosystem.

[2] Shabat S.K.B., Sasson G., Doronfaigenboim A., Durman T., Yaacoby S., Miller M.E.B., White B.A., Shterzer N., Mizrahi I. (2016). Specific microbiome-dependent mechanisms underlie the energy harvest efficiency of ruminants. ISME J..

[3] Zhou M., Peng Y.J., Chen Y., Klinger C.M., Oba M., Liu J.X., Guan L.L. (2018). Assessment of microbiome changes after rumen transfaunation: Implications on improving feed efficiency in beef cattle. Microbiome.

[4] Li F., Guan L.L. (2017). Metatranscriptomic Profiling Reveals Linkages between the Active Rumen Microbiome and Feed Efficiency in Beef Cattle. Appl. Environ. Microbiol..

[5] Kumar S., Indugu N., Vecchiarelli B., Pitta D.W. (2015). Associative patterns among anaerobic fungi, methanogenic archaea, and bacterial communities in response to changes in diet and age in the rumen of dairy cows. Front. Microbiol..

[6] Yang C., Takahashi T., Horiguchi K. (2009). Effects of addition of food by-products on the fermentation quality of a total mixed ration with whole crop rice and its digestibility, preference, and rumen fermentation in sheep. Anim. Feed. Sci. Technol..

[7] Wang F., Nishino N. (2008). Ensiling of soybean curd residue and wet brewers grains with or without other feeds as a total mixed ration. J. Dairy Sci..

[8] Wang J., Wang J., Bu D., Guo W., Song Z., Zhang J. (2010). Effect of storing total mixed rations anaerobically in bales on feed quality. Anim. Feed Sci. Technol..

[9] Han H., Ogata Y., Yamamoto Y., Nagao S., Nishino N. (2014). Identification of lactic acid bacteria in the rumen and feces of dairy cows fed total mixed ration silage to assess the survival of silage bacteria in the gut. J. Dairy Sci..

[10] Yuan X., Guo G., Wen A., Desta S.T., Wang J., Wang Y., Shao T. (2015). The effect of different additives on the fermentation quality, in vitro digestibility and aerobic stability of a total mixed ration silage. Anim. Feed Sci. Technol..

[11] Wang C., Nishino N. (2010). Presence of sourdough lactic acid bacteria in commercial total mixed ration silage as revealed by denaturing gradient gel electrophoresis analysis. Lett. Appl. Microbiol..

[12] Liu Q.H., Xiang-Yu L.I., Desta S.T., Zhang J.G., Shao T. (2016). Effects of Lactobacillus plantarum and fibrolytic enzyme on the fermentation quality and in vitro digestibility of total mixed rations silage including rape straw. J. Integr. Agric..

[13] Nishino N., Wada H., Yoshida M., Shiota H. (2004). Microbial counts, fermentation products, and aerobic stability of whole crop corn and a total mixed ration ensiled with and without inoculation of Lactobacillus casei or Lactobacillus buchneri. J. Dairy Sci..

[14] Li Ying H., Borjigin N., Yu Z. (2017). Effect of inoculants and fibrolytic enzymes on the fermentation characteristics, in vitro digestibility and aflatoxins accumulation of whole-crop corn silage. Grassl. Sci..

[15] Cao Y., Takahashi T., Horiguchi K.-i., Yoshida N., Cai Y. (2010). Methane emissions from sheep fed fermented or non-fermented total mixed ration containing whole-crop rice and rice bran. Anim. Feed Sci. Technol..

[16] Cao Y., Zang Y., Jiang Z., Han Y., Hou J.J., Liu H., Zhong R., Fang J., Zhang A., Yoshida N. (2016). Fermentation quality and nutritive value of fresh and fermented total mixed rations containing Chinese wildrye or corn stover. Grassl. Sci..

[17] Kung L., Muck R. Animal response to silage additives. Proceedings of the Conference on Silage: Field to Feedbunk.

[18] Kung L. Potential Factors that May Limit the Effectiveness of Silage Additives. Proceedings of the XV International Silage Conference.

[19] Bryant M.P., Burkey L.A. (1953). Numbers and some predominant groups of bacteria in the rumen of cows fed different rations. J. Dairy Sci..

[20] Warner A.C.I. (1962). Some Factors Influencing the Rumen Microbial Population. J. Gen. Microbiol..

[21] Leedle J.A., Bryant M.P., Hespell R.B. (1982). Diurnal variations in bacterial numbers and fluid parameters in ruminal contents of animals fed low- or high-forage diets. Appl. Environ. Microbiol..

[22] Weinberg Z.G., Muck R.E., Weimer P.J. (2010). The survival of silage inoculant lactic acid bacteria in rumen fluid. J. Appl. Microbiol..

[23] Mohammed R., Stevenson D., Beauchemin K., Muck R., Weimer P. (2012). Changes in ruminal bacterial community composition following feeding of alfalfa ensiled with a lactic acid bacterial inoculant. J. Dairy Sci..

[24] Contreras-Govea F.E., Muck R.E., Mertens D.R., Weimer P.J. (2011). Microbial inoculant effects on silage and in vitro ruminal fermentation, and microbial biomass estimation for alfalfa, bmr corn, and corn silages. Anim. Feed Sci. Technol..

[25] Weimer P.J. (2014). Redundancy, resilience, and host specificity of the ruminal microbiota: Implications for engineering improved ruminal fermentations. Front. Microbiol..

[26] Malmuthuge N., Guan L.L. (2017). Understanding host-microbial interactions in rumen: Searching the best opportunity for microbiota manipulation. J. Anim. Sci. Biotechnol..

[27] Boston R.C., Fox D.G., Sniffen C., Janczewski E., Munson R., Chalupa W. (2000). The conversion of a scientific model describing dairy cow nutrition and production to an industry tool: The CPM Dairy project. Modelling Nutrient Utilization in Farm Animals.

[28] Wang B., Mao S.Y., Yang H.J., Wu Y.M., Wang J.K., Li S.L., Shen Z.M., Liu J.X. (2014). Effects of alfalfa and cereal straw as a forage source on nutrient digestibility and lactation performance in lactating dairy cows. J. Dairy Sci..

[29] Hao X., Gao H., Wang X., Zhang G., Zhang Y. (2017). Replacing alfalfa hay with dry corn gluten feed and Chinese wild rye grass: Effects on rumen fermentation, rumen microbial protein synthesis, and lactation performance in lactating dairy cows. J. Dairy Sci..

[30] Zhang J., Shi H., Wang Y., Li S., Cao Z., Ji S., He Y., Zhang H. (2017). Effect of dietary forage to concentrate ratios on dynamic profile changes and interactions of ruminal microbiota and metabolites in Holstein heifers. Front. Microbiol..

[31] Rius A.G., Kittelmann S., Macdonald K.A., Waghorn G.C., Janssen P.H., Sikkema E. (2012). Nitrogen metabolism and rumen microbial enumeration in lactating cows with divergent residual feed intake fed high-digestibility pasture. J. Dairy Sci..

[32] Caporaso J.G., Kuczynski J., Stombaugh J., Bittinger K., Bushman F.D., Costello E.K., Fierer N., Peña A.G., Goodrich J.K., Gordon J.I. (2010). QIIME allows analysis of high-throughput community sequencing data. Nat. Methods.

[33] Edgar R.C. (2010). Search and clustering orders of magnitude faster than BLAST. Bioinformatics.

[34] Pruesse E., Quast C., Knittel K., Fuchs B.M., Ludwig W., Peplies J., Glöckner F.O. (2007). SILVA: A comprehensive online resource for quality checked and aligned ribosomal RNA sequence data compatible with ARB. Nucleic Acids Res..

[35] Wolf M., Koetschan C., Müller T. (2014). ITS2, 18S, 16S or any other RNA—Simply aligning sequences and their individual secondary structures simultaneously by an automatic approach. Gene.

[36] Kõljalg U., Larsson K.H., Abarenkov K., Nilsson R.H., Alexander I.J., Eberhardt U., Erland S., Høiland K., Kjøller R., Larsson E. (2005). UNITE: A database providing web-based methods for the molecular identification of ectomycorrhizal fungi. New Phytol..

[37] Segata N., Izard J., Waldron L., Gevers D., Miropolsky L., Garrett W.S., Huttenhower C. (2011). Metagenomic biomarker discovery and explanation. Genome Biol..

[38] Reynolds C., Kristensen N.B. (2008). Nitrogen recycling through the gut and the nitrogen economy of ruminants: An asynchronous symbiosis. J. Anim. Sci..

[39] Brito A.F., Broderick G.A., Reynal S.M. (2007). Effects of Different Protein Supplements on Omasal Nutrient Flow and Microbial Protein Synthesis in Lactating Dairy Cows1. J. Dairy Sci..

[40] Wang Y., Yu Q., Wang X., Song J., Lambo M.T., Huang J., He P., Li Y., Zhang Y. (2023). Replacing alfalfa hay with industrial hemp ethanol extraction byproduct and Chinese wildrye hay: Effects on lactation performance, plasma metabolites, and bacterial communities in Holstein cows. Front. Vet. Sci..

[41] Hristov A., Ropp J. (2003). Effect of dietary carbohydrate composition and availability on utilization of ruminal ammonia nitrogen for milk protein synthesis in dairy cows. J. Dairy Sci..

[42] Li Y., Gao J., Lv J., Lambo M.T., Wang Y., Wang L., Zhang Y. (2023). Replacing soybean meal with high-oil pumpkin seed cake in the diet of lactating Holstein dairy cows modulated rumen bacteria and milk fatty acid profile. J. Dairy Sci..

[43] Miyaji M., Inoue H., Kawaide T., Tohno M., Kamiya Y., Nonaka K. (2017). Effects of conservation method and crushing method of rice grain on rumen fermentation and nutrient digestibility in steers. Anim. Feed Sci. Technol..

[44] Russell J.B., Wallace R.J. (1997). Energy-Yielding and Energy-Consuming Reactions.

[45] Woodford S.T., Murphy M.R. (1988). Effect of forage physical form on chewing activity, dry matter intake, and rumen function of dairy cows in early lactation. J. Dairy Sci..

[46] Jami E., Israel A., Kotser A., Mizrahi I. (2013). Exploring the bovine rumen bacterial community from birth to adulthood. ISME J..

[47] Henderson G., Cox F., Ganesh S., Jonker A., Young W., Collaborators G.R.C., Abecia L., Angarita E., Aravena P., Arenas G.N. (2015). Rumen microbial community composition varies with diet and host, but a core microbiome is found across a wide geographical range. Sci. Rep..

[48] Lan M., Yang B., Hu X., Yang L., Liu J., Yu Z., Wang J. (2018). Comparative Analysis of the Microbiota Between Sheep Rumen and Rabbit Cecum Provides New Insight Into Their Differential Methane Production. Front. Microbiol..

[49] Shen H., Lu Z., Chen Z., Wu Y., Shen Z. (2016). Rapid Fermentable Substance Modulates Interactions between Ruminal Commensals and Toll-Like Receptors in Promotion of Immune Tolerance of Goat Rumen. Front. Microbiol..

[50] Derrien M., Van Baarlen P., Hooiveld G., Norin E., Muller M., de Vos W. (2011). Modulation of mucosal immune response, tolerance, and proliferation in mice colonized by the mucin-degrader Akkermansia muciniphila. Front. Microbiol..

[51] Png C.W., Lindén S.K., Gilshenan K.S., Zoetendal E.G., McSweeney C.S., Sly L.I., McGuckin M.A., Florin T.H. (2010). Mucolytic bacteria with increased prevalence in IBD mucosa augment in vitro utilization of mucin by other bacteria. Am. J. Gastroenterol..

[52] Elie J., Itzhak M. (2012). Composition and similarity of bovine rumen microbiota across individual animals. PLoS ONE.

[53] Zhou Z., Fang L., Meng Q., Li S., Chai S., Liu S., Schonewille J.T. (2017). Assessment of Ruminal Bacterial and Archaeal Community Structure in Yak (*Bos grunniens*). Front. Microbiol..

[54] Neubauer V., Petri R., Humer E., Kröger I., Mann E., Reisinger N., Wagner M., Zebeli Q. (2018). High-grain diets supplemented with phytogenic compounds or autolyzed yeast modulate ruminal bacterial community and fermentation in dry cows. J. Dairy Sci..

[55] Mao S.Y., Huo W.J., Zhu W.Y. (2015). Microbiome–metabolome analysis reveals unhealthy alterations in the composition and metabolism of ruminal microbiota with increasing dietary grain in a goat model. Environ. Microbiol..

[56] Sandra K., Henning S., Walters W.A., Clemente J.C., Rob K., Gordon J.I., Janssen P.H. (2013). Simultaneous amplicon sequencing to explore co-occurrence patterns of bacterial, archaeal and eukaryotic microorganisms in rumen microbial communities. PLoS ONE.

[57] Hook S.E., Steele M.A., Northwood K.S., Dijkstra J., France J., Wright A.G., Mcbride B.W. (2011). Impact of subacute ruminal acidosis (SARA) adaptation and recovery on the density and diversity of bacteria in the rumen of dairy cows. FEMS Microbiol. Ecol..

[58] Dieho K., Bogert B.V.D., Henderson G., Bannink A., Ramiro-Garcia J., Smidt H., Dijkstra J. (2017). Changes in rumen microbiota composition and in situ degradation kinetics during the dry period and early lactation as affected by rate of increase of concentrate allowance. J. Dairy Sci..

[59] Goad D.W., Goad C.L., Nagaraja T.G. (1998). Ruminal microbial and fermentative changes associated with experimentally induced subacute acidosis in steers. J. Anim. Sci..

[60] Hristov A.N., Ivan M., Rode L.M., McAllister T.A. (2001). Fermentation characteristics and ruminal ciliate protozoal populations in cattle fed medium- or high-concentrate barley-based diets. J. Anim. Sci..

[61] Danielsson R., Dicksved J., Sun L., Gonda H., Müller B., Schnürer A., Bertilsson J. (2017). Methane Production in Dairy Cows Correlates with Rumen Methanogenic and Bacterial Community Structure. Front. Microbiol..

[62] Leahy S.C., Kelly W.J., Ronimus R.S., Wedlock N., Altermann E., Attwood G.T. (2013). Genome sequencing of rumen bacteria and archaea and its application to methane mitigation strategies. Animal.

[63] Zhu Z., Han Y., Ding Y., Zhu B., Song S., Xiao H. (2021). Health effects of dietary sulfated polysaccharides from seafoods and their interaction with gut microbiota. Compr. Rev. Food Sci. Food Saf..

[64] Bui T.P.N., Schols H.A., Jonathan M., Stams A.J.M., de Vos W.M., Plugge C.M. (2019). Mutual Metabolic Interactions in Co-cultures of the Intestinal Anaerostipes rhamnosivorans With an Acetogen, Methanogen, or Pectin-Degrader Affecting Butyrate Production. Front. Microbiol..

[65] Clemmons B.A., Powers J.B., Campagna S.R., Seay T.B., Embree M.M., Myer P.R. (2020). Rumen fluid metabolomics of beef steers differing in feed efficiency. Metabolomics.

[66] Arshad U., Zenobi M.G., Staples C.R., Santos J.E.P. (2020). Meta-analysis of the effects of supplemental rumen-protected choline during the transition period on performance and health of parous dairy cows. J. Dairy Sci..

[67] Connor E.E. (2015). Invited review: Improving feed efficiency in dairy production: Challenges and possibilities. Animal.

